# Comparative Resistance of Bacterial Foodborne Pathogens to Non-thermal Technologies for Food Preservation

**DOI:** 10.3389/fmicb.2016.00734

**Published:** 2016-05-20

**Authors:** Guillermo Cebrián, Pilar Mañas, Santiago Condón

**Affiliations:** Tecnología de los Alimentos, Facultad de Veterinaria de Zaragoza, Instituto Agroalimentario de Aragón – IA2 – (Universidad de Zaragoza-CITA), ZaragozaSpain

**Keywords:** non-thermal technologies, food preservation, ultrasound, manosonication, pulsed electric fields, high hydrostatic pressure, UV, foodbone pathogens

## Abstract

In this paper the resistance of bacterial foodborne pathogens to manosonication (MS), pulsed electric fields (PEFs), high hydrostatic pressure (HHP), and UV-light (UV) is reviewed and compared. The influence of different factors on the resistance of bacterial foodborne pathogens to these technologies is also compared and discussed. Only results obtained under harmonized experimental conditions have been considered. This has allowed us to establish meaningful comparisons and draw significant conclusions. Among the six microorganisms here considered, *Staphyloccocus aureus* is the most resistant foodborne pathogen to MS and HHP and *Listeria monocytogenes* to UV. The target microorganism of PEF would change depending on the treatment medium pH. Thus, *L. monocytogenes* is the most PEF resistant microorganism at neutral pH but Gram-negatives (*Escherichia coli*, *Salmonella* spp., *Cronobacter sakazakii, Campylobacter jejuni*) would display a similar or even higher resistance at acidic pH. It should be noted that, in acidic products, the baroresistance of some *E. coli* strains would be comparable to that of *S. aureus*. The factors affecting the resistance of bacterial foodborne pathogens, as well as the magnitude of the effect, varied depending on the technology considered. Inter- and intra-specific differences in microbial resistance to PEF and HHP are much greater than to MS and UV. Similarly, both the pH and a_w_ of the treatment medium highly condition microbial resistance to PEF and HHP but no to MS or UV. Growth phase also drastically affected bacterial HHP resistance. Regarding UV, the optical properties of the medium are, by far, the most influential factor affecting its lethal efficacy. Finally, increasing treatment temperature leads to a significant increase in lethality of the four technologies, what opens the possibility of the development of combined processes including heat. The appearance of sublethally damaged cells following PEF and HHP treatments could also be exploited in order to design combined processes. Further work would be required in order to fully elucidate the mechanisms of action of these technologies and to exhaustively characterize the influence of all the factors acting before, during, and after treatment. This would be very useful in the areas of process optimization and combined process design.

## Introduction

The food industry is showing growing interest in developing alternative microbial inactivation methods capable of avoiding the undesirable effects that traditional technologies such as heating or acidification cause on foods (Mañas and Pagán, 2005). Thus, a number of different methods including pulsed electric fields (PEFs), high hydrostatic pressure (HHP), ultrasound (US), and UV light (UV) have been proposed as possible alternatives to traditional technologies.

A wide amount of data on the resistance of different microbial species of relevance for food safety and stability to each of the four technologies that are to be reviewed in this paper (US, PEF, UV, and HHP) is available. However, different types of equipment, matrices, and experimental conditions applied in a number of studies and/or laboratories (for example the microorganisms’ physiological state, differing treatment parameters and recovery conditions) make it difficult to classify the relative resistance of different microbial species to each of these technologies and almost impossible to establish meaningful comparisons among the latter. Unfortunately, if we want to find an application for a new food preservation technology, we initially need such comparisons to help us identify which products could be processed by it, or, generally speaking, which is the most suitable technology for a specific product.

The aim of this paper is to review and compare the resistance of bacterial foodborne pathogens to four non-thermal technologies for food preservation: US, PEF, HHP, and UV. The influence of different factors on the resistance of bacterial foodborne pathogens to these technologies will be also compared and discussed. For comparative purposes, in some cases we will also include microbial heat resistance data. As pointed out above, a basic pre-requisite for establishing meaningful comparisons and drawing significant conclusions is the harmonization of the experimental conditions that reigned in all studies under review. In this regard, great advantage and interest can be found in the comparison of results obtained by a single research group using the same strains as well as the same protocols for obtaining suspensions and for recovering treated cells -all evaluated by the same matrices and using identical equipment-. For this reason, the present review will focus on the data obtained by the New Technologies for Food Preservation group (DGA-A20) at the University of Zaragoza along the last 25 years, since all its results were obtained following standardized protocols: thus, they are directly comparable with one another.

## Non-Thermal Technologies for Microbial Inactivation in Food

In this review, four of the most promising non-thermal food preservation technologies will be considered: US, PEF, HHP, and UV.

### Ultrasound-Manosonication

Ultrasound consists in the use of sonic waves with frequencies exceeding 16–18 kHz, which lie above the threshold of human hearing. It is one of the new microbial inactivation technologies suggested as an alternative to current heat treatments (Condón et al., 2011). Nevertheless, one should note that the bactericidal effect of ultrasound has been known since the early 20th century (Harvey and Loomis, 1929).

It is generally acknowledged that the lethality of high power ultrasound (20–40 kHz) on microbial cells is due to a phenomenon called transient cavitation (Kinsloe et al., 1954; Davies, 1959; Raso et al., 1998d; Condón et al., 2005). As a consequence of the implosion of bubbles generated during transient cavitation, molecules violently collide, thereby producing shock waves which, in turn, lead to spots of extremely high temperature (5000°C) and pressure (100 MPa; Suslick, 1988). The high temperatures and pressures produced in the bubbles’ implosion spots can also provoke the dissociation of water molecules into OH radicals and H atoms (Suslick, 1990). Although, it was initially hypothesized that the “hot spots” and reactive species generated from water sonolysis might contribute to ultrasound’s lethal effect, most authors now agree that the shock waves produced during transient cavitation are probably the main or sole mechanism responsible for microbial inactivation (Davies, 1959; Raso, 1995; Pagán, 1997; Valero et al., 2007; Condón et al., 2011). Furthermore, various studies have shown that ultrasound destroys cellular envelopes (Guerrero et al., 2001; Condón et al., 2011).

According to most data reported, the germ-killing efficacy of ultrasound is still relatively low in room conditions and could only become an actual alternative to current heat treatments under special circumstances (Alzamora et al., 2011). Therefore, most investigators have tried to improve the procedure’s efficacy either by increasing cavitation intensity, or by designing combined processes that would enhance the effect of ultrasound (Ordóñez et al., 1984, 1986; Sala et al., 1992; Raso et al., 1998d,e; Arce-García et al., 2002; Lee et al., 2003; Guerrero et al., 2005; López-Malo et al., 2005). In this review we will focus on *manosonication* (MS), a process designed and patented by our group (MTS, Spanish Patent No 9200686); MS probably represents the most promising approach to non-thermal food pasteurization involving ultrasound.

### Pulsed Electric Fields

One of the most promising new technologies for microbial inactivation is PEF, consisting in the application of short duration (1–100 μs) high electric field pulses (10–50 kV/cm) to food placed between two electrodes (Heinz et al., 2001), PEF is capable of inactivating microorganisms while causing little changes in the sensory and nutritional quality of foodstuffs (Raso and Barbosa-Cánovas, 2003).

Similarly to ultrasound, PEF’s main targets are cellular envelopes (Mañas and Pagán, 2005), since PEFs are capable of temporarily or permanently permeabilizing cell membranes -a phenomenon known as “electropermeabilization” or “electroporation.” The complex phenomenon of membrane electroporation has been widely investigated, and several theories have been proposed to explain it (Zimmermann, 1986; Tsong, 1991; Kinosita et al., 1992; Ho and Mittal, 1996; Weaver and Chizmadzhev, 1996; Barbosa-Cánovas et al., 1999; Pavlin et al., 2007). No clear evidence has yet been found, however, of the underlying mechanism of membrane permeabilization on a molecular level (Pagán and Mañas, 2006).

### High Hydrostatic Pressure

High hydrostatic pressure technology consists in the application of pressures ranging from 100 to 15,000 MPa to food products (Ledward, 1995). Similarly to ultrasound and PEF, its objective is to inactivate pathogenic and spoilage microorganisms without affecting food quality (Smelt, 1998; Mañas and Pagán, 2005; Patterson, 2005). The first studies on the lethal effect of HHP were conducted at the end of the 19th century, but only in recent years have commercial applications of this procedure started seeing the light of day. As opposed to the other new technologies reviewed in this paper, HHP is already being applied commercially for the preservation of a wide range of food products.

Most authors agree that microbial HHP inactivation is a multi-target process (Mañas and Pagán, 2005). As in MS and PEF, the membrane would be a key target; however, in some cases, additional damaging events also seem to be necessary in order to kill bacteria -events such as extensive solute loss during pressurization, protein coagulation, key enzyme inactivation and ribosome conformational changes, along with impaired recovery mechanisms (Mañas and Pagán, 2005)-. In this sense, it has also been hypothesized that HHP might affect cytoplasmic or membrane enzymes, thereby disturbing cellular metabolism and inducing the generation of endogenous reactive oxygen species (ROS). This would lead to an accumulation of oxidative damage and cell death (Aertsen et al., 2005).

### Ultraviolet Light

Although ultraviolet (UV) light irradiation has been used traditionally for air, surface, and water decontamination, only recently has the food industry developed an interest in its possible application to the hygienization of liquid foods and the surfaces of solid foods (Gayán et al., 2014a).

Corresponding to the portion of the electromagnetic spectrum ranging from 200 to 400 nm, UV light is divided into three regions: short-wave ultraviolet (UV-C) from 200 to 280 nm; medium-wave UV (UV-B) from 280 to 320 nm; and long-wave UV (UV-A) from 320 to 400 nm. UV-C is the most germicidal region, and the peak of maximum effectiveness can be found at wavelengths of ca. 260–265 nm, corresponding to the peak of maximum DNA absorption (Kowalski, 2009). Thus, although other cellular components such as proteins can also undergo damage, the effects of UV light on genetic material are the main factor responsible for this technology’s capacity for microbial inactivation (Gayán et al., 2014a).

### Microbial Inactivation by MS, PEF, HHP, and UV: Potential Applications and Inactivation Kinetics

Microorganisms vary widely in their resistance to the four non-thermal technologies for food preservation here reviewed. Yeast and vegetative mold are considerably more MS-, PEF-, and HHP- sensitive than prokaryotic cells (Raso et al., 1994, Raso et al., 1998b; Somolinos et al., 2008b; Puértolas et al., 2009) but they are usually more resistant than vegetative bacteria to UV (Gayán, 2014; Gayán et al., 2014a). On the other hand, bacterial spores are, as a general rule, the most resistant micro-organisms to physical stresses (Mañas and Pagán, 2005). This holds true for the four technologies reviewed herein, as well as for heat. Published data demonstrate that PEF does not affect the viability of spores (Pagán et al., 1998; Raso et al., 1998a,b; Mañas and Pagán, 2005; Álvarez et al., 2006a) and that they are also extremely resistant to HHP: they possess the capacity to withstand up to 1000 MPa for extended treatment intervals, unless they are in a state of germination (Cheftel, 1995; Raso et al., 1998c; Ramos, 2016). Conversely, MS and UV treatments could both be used as food sterilization methods, since they are capable of inactivating spores. Although, spores are more resistant to MS and UV than vegetative cells it should be noted that the difference in resistance between spores and vegetative cells to MS (10-fold) and UV (3- to 50-fold) is negligible when compared with the 10^7^ times difference in resistance to heat (Setlow, 2001; Condón et al., 2011; Gayán et al., 2013a). Finally, data in the bibliography indicate that virus would be even more resistant than spores to UV (Gayán et al., 2014a) and that their HHP resistance would be quite heterogeneous; but for some of them (such as human rotavirus and hepatitis-A virus) it would be comparable to that of vegetative bacteria (Smelt, 1998; Khadre and Yousef, 2002; Kingsley et al., 2002). On the other hand, viruses would display a high resistance to PEF (Khadre and Yousef, 2002). To the best of our knowledge, no data are available regarding the resistance of viruses to ultrasound.

Since, as pointed out above, not all the technologies reviewed herein are useful for sterilization purposes, this review will focus only on those species which are of relevance in food pasteurization processes. According to EFSA data (EFSA, 2014) the most frequent bacteria responsible for cases of human illness and/or foodborne outbreaks are *Campylobacter* spp. and *Salmonella* spp. On the other hand, *Staphylococcus aureus* is the foremost cause of foodborne intoxications. Many other species are capable of causing foodborne illness in humans, but *Listeria monocytogenes* and certain *Escherichia coli* strains stand out among the other due to their high mortality rates. Finally, *Cronobacter sakazakii* is an emergent pathogen that has recently attracted a great deal of interest after having caused several outbreaks and cases of neonatal infection (Arroyo et al., 2009). Thus, where possible, comparisons presented in this work will be based on resistance data obtained for these species, although in some cases data for *Campylobacter* is not available, and corresponding comments will be included only in the text.

Regarding the kinetics of microbial inactivation by these technologies it can be concluded that MS survival curves tend to display a linear profile (Condón et al., 2011); PEF survival curves usually feature tails (Álvarez et al., 2000, 2002, 2003a,c,d,e; Raso et al., 2000; Gómez et al., 2005; Cebrián et al., 2007, 2009; Arroyo et al., 2010a; Sagarzazu et al., 2010b; Saldaña et al., 2010a,b,c) but occurrence of shoulders in PEF survival curves is much more unusual -in our research group, shoulders have only been found in PEF survival curves of *C. sakazakii* cells treated in media with reduced a_w_ (Arroyo et al., 2010a)-, and UV curves usually feature shoulders (Gayán et al., 2011, 2012a,b,c, 2014b, 2015; Arroyo et al., 2012b). The profile of HHP survival curves varies widely depending on the strain, on the treatment and on medium conditions: it can be linear, concave, convex or even sigmoid (Mañas and Pagán, 2005; Somolinos et al., 2008b; Cebrián et al., 2009, 2010a; Arroyo et al., 2011a; Ramos, 2016).

Deviations from linearity in survival curves have very relevant practical implications. On the one hand, new mathematical models are required to describe the observed kinetics. Indeed, many models and equations have been developed and applied for this purpose, but a detailed review there of would be beyond the scope of this paper. On the other hand, this wide variety of models makes it very difficult to establish meaningful comparisons. To address this hindrance, we will use the time or dose required to inactivate 4 log cycles (*4D* value) as a parameter to compare the resistance of bacterial foodborne pathogens to the different technologies reviewed herein.

## Comparative Resistance of Foodborne Pathogens to MS, PEF, HHP, and UV

**FIGURE 1:**
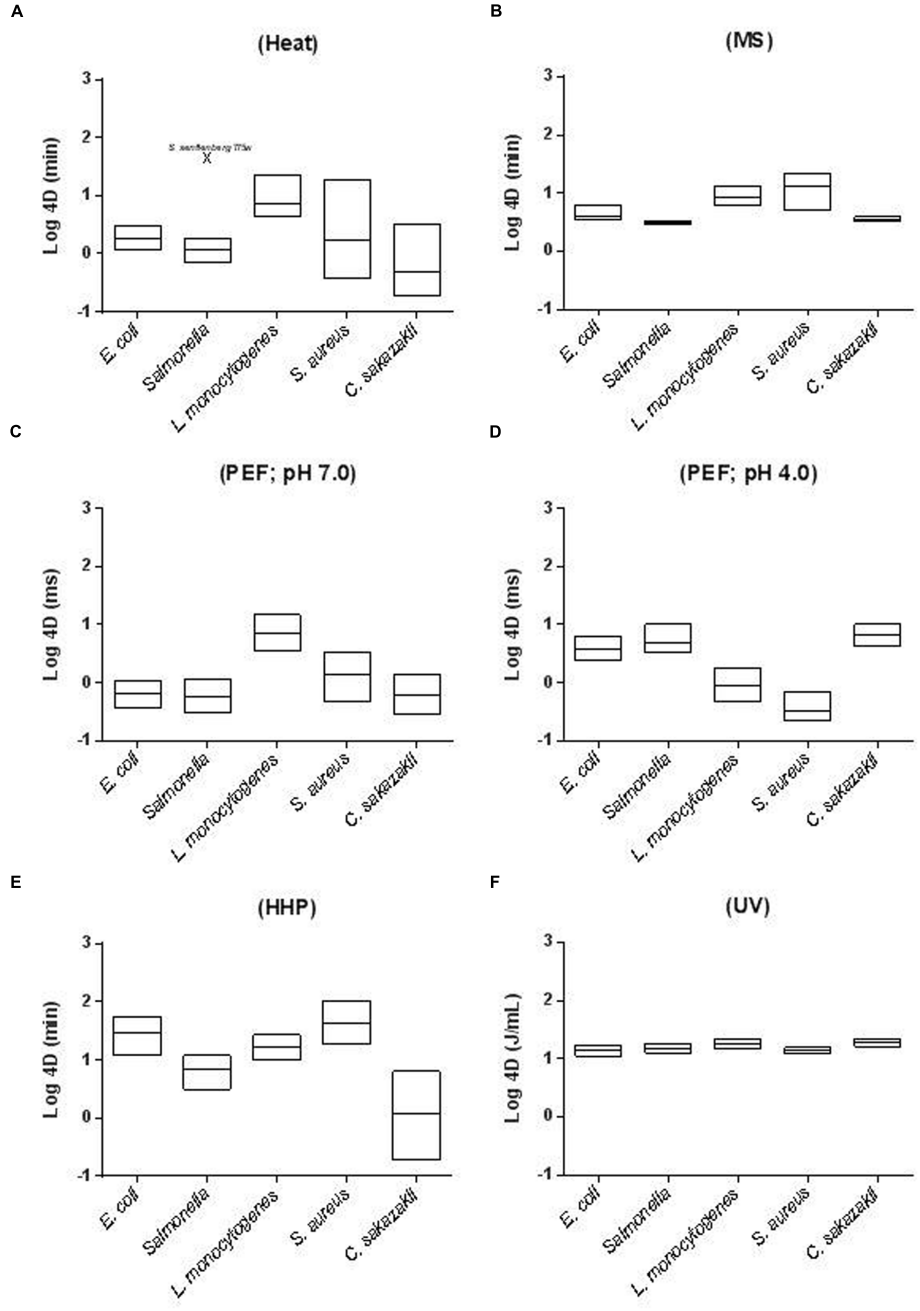
**Inter- and intra-specific differences in resistance of different foodborne pathogens to **(A)** heat (58°C; McIlvaine buffer pH 7.0); **(B)** MS (117 μm; 200 KPa; McIlvaine buffer pH 7.0); **(C)** PEF (26 kV/cm; ≈4 μs exponential waveform pulses; McIlvaine buffer pH 7.0 and 2 mS cm^-1^); **(D)** PEF (26 kV/cm; ≈4 μs exponential waveform pulses; McIlvaine buffer pH 4.0 and 2 mS cm^-1^); **(E)** HHP (450 MPa; McIlvaine buffer pH 7.0) and **(F)** UV (McIlvaine buffer pH 7.0 and an absorption coefficient of 11.0 cm^-1^).** In the figure, floating bars indicate the *4D* values for the most resistant (maximum) and less resistant (minimum) strain of each species. The line inside the bar corresponds to the average *4D* value for each species. See text for references.

The relative resistance of bacterial foodborne pathogens to MS (B), PEF (C,D), HHP (E), and UV (F) when treated in Mc-Ilvaine buffer of pH 7.0 (except for figure D, where the data comes from cells treated at pH 4.0) is depicted in **Figure 1**. Relative resistance to heat is also included for comparative purposes (A). In the figure, floating bars indicate the *4D* values for the most resistant (maximum) and least resistant (minimum) strain of each species. Thus, bar length reflects the intra-specific differences in resistance. The line inside the bar corresponds to the average *4D* value for each species.

As can be observed *S. aureus* is the most MS-resistant pathogen and *Salmonella enterica* and *C. sakazakii* the most sensitive ones (Pagán et al., 1999a,c; Mañas et al., 2000a; Álvarez et al., 2003b, 2006b; Rodríguez-Calleja et al., 2006; Arroyo et al., 2010b). Both inter- and intra-specific differences in resistance to MS were very small. Thus, inter-specific *4D_MS_* resistance values varied less than 3.5-fold and intra-specific less than 4.5-fold (**Figure 1B**). Inter-specific differences in resistance were even lower for UV treatments (less than 1.5-fold). In this case, *L. monocytogenes* appeared as the most resistant species and *S. enterica* again as the most sensitive one. Intra-specific differences in resistance to UV were also very low, especially when compared with intra-specific resistance to other technologies; nevertheless, those differences are of a similar or even greater magnitude than the inter-specific differences in resistance to UV (Gayán et al., 2011, 2012a,b,c, 2014b, 2015; Arroyo et al., 2012b).

Again, *S. aureus* appears as the most baro-resistant microorganism and *Salmonella* and *C. sakazakii* as the least baro-resistant. It should be highlighted that some particular *E. coli* strains are very HHP-resistant (Somolinos et al., 2008b; Cebrián et al., 2009, 2010a; Sagarzazu et al., 2010a; Arroyo et al., 2011a; Espina et al., 2013; Ramos, 2016). Although not included in the figure, *Campylobacter* is the least HHP-resistant microorganism among those studied (Sagarzazu et al., 2010a). It should be remarked that, conversely to MS and UV, resistance of foodborne pathogens to HHP varies widely. Thus, both inter- and intra-specific differences in resistance to HHP (*4D* values) are greater than 30-fold (**Figure 1E**).

Regarding PEF, it should be noted that, certain treatment conditions not only modify bacterial PEF resistance, but do so in different directions (see Treatment Medium pH). Because of this, **Figure 1** includes the *4D_PEF_* values at both pH 7.0 (1C) and 4.0. (1D). The most resistant species to PEF at pH 7.0 would be *L. monocytogenes*. Conversely, *E. coli*, *C. sakazakii*, and *Salmonella* would be the most resistant at pH 4.0 (**Table 1**). Nevertheless, it should be noted that from the data of Saldaña et al. (2010a,b) it can be deduced that the PEF resistance of some *L. monocytogenes* strains would be comparable to that of Gram-negatives. Regarding *Campylobacter*, data obtained by Sagarzazu et al. (2010b) demonstrate that whereas *Campylobacter jejuni* is the least HHP and heat resistant microorganism, its PEF resistance would be comparable to that of the other three Gram-negatives evaluated. Although some exceptions have been published, Rodríguez-Calleja et al. (2006) demonstrated that intra-specific differences in PEF resistance are also very low. Conversely, inter-specific differences are much larger (García et al., 2005a,b, 2007; Cebrián et al., 2007; Arroyo et al., 2010a; Somolinos et al., 2010b). Thus, from the figure it can be concluded that pathogen resistance may vary up to 12-fold at pH 7.0 and more than 18-fold at 4.0.

**Table 1:** Most resistant foodborne pathogen/s to heat, manosonication (MS), pulsed electric fields (PEFs), high hydrostatic pressure (HHP), and UV-light (UV) when treated at different treatment medium pH and a_w_.

	Heat	MS	PEF	HHP	UV
pH 7.0 a_w_>0.99	*S. senftemberg 775 W*	*S. aureus*	*L. monocytogenes*	*S. aureus*	L. monocytogenes*
pH 4.0 a_w_>0.99	S. aureus** S. senftemberg 775 W	*S. aureus*	Gram-negatives***	S. aureus**** E. coli	L. monocytogenes*
pH 7.0 a_w_=0.96	*L. monocytogenes*	*S. aureus*	*L. monocytogenes*	*S. aureus*	L. monocytogenes*

See text for references. *Intra-specific differences in resistance to UV usually surpass inter-specific ones. **Hassani et al. (2006); Cebrián (2009). ***Resistance to PEF at pH 4.0 of *E. coli, S. typhimurium*, *C. sakazakii*, and *C. jejuni* is comparable. Some authors indicate that the PEF resistance of some strains of *L. monocytogenes* at pH 4.0 would be comparable to that of Gram-negatives (Saldaña et al., 2010a,b). ****See text (see Treatment Medium pH).

In any case, inter- and intra-specific differences in resistance to heat exceed those to other technologies, in general (**Figure 1A**). Large intra-specific differences in resistance to heat are especially evident for *S. aureus*, *C. sakazakii*, and *Salmonella* (Mañas et al., 2001b, 2003; Rodríguez-Calleja et al., 2006; Cebrián et al., 2007; Arroyo et al., 2009). Nevertheless, it should be noted that for *Salmonella* these differences are smaller if *S. senftenberg* 775W is excluded from the analysis. This particular strain displays a heat resistance 10–100 times greater than any other of its genera. Regarding the most and least resistant pathogens to heat, *S. senftenberg* 775W, *L. monocytogenes* and some strains of *S. aureus* would be the most heat resistant, whereas *Campylobacter* would be even the least heat-resistant one (Sagarzazu et al., 2010b).

From the data presented herein, one can conclude that the most resistant microorganism to MS and HHP in terms of average species resistance would be *S. aureus*. *L. monocytogenes* would be the most resistant to UV, to PEF at pH 7.0 and to heat -along with *S. senftenberg* 775W-. Finally, *Salmonella*, *E. coli*, and *C. sakazakii* would be the most PEF-resistant microorganisms when treatment pH is lowered to 4.0 (**Table 1**). As will be discussed later, a multitude of factors affect microbial resistance; thus, these conclusions should be viewed with care. They are based on the comparison of resistance of stationary growth phase cells grown in Tryptic Soy Broth (TSB) at 37°C, treated in McIlvaine buffer of pH 7.0 and recovered in Tryptic Soy Agar (TSA), also at 37°C. However, if growth, treatment or recovery conditions are modified, the most resistant species/strain to a particular technology can change. Thus, in the review presented in the next section about factors affecting microbial resistance to MS, PEF, HHP and UV, it will be also indicated if the modification of any experimental condition would lead to changes in the classification that we have presented (**Table 1**). From these data it can also be deduced Gram-positive pathogens -*S. aureus* and *L. monocytogenes*- are the ones displaying the highest resistance to MS, PEF, HHP, and UV in most scenarios. The greater rigidity of Gram-positive envelopes is regarded as the main reason for their increased resistance to ultrasound and HHP (Mañas and Pagán, 2005), since the mode of action of both technologies involves physical damages in the envelopes. Additionally, the size and shape of *S. aureus* might explain its increased resistance to MS and HHP -as compared to *L. monocytogenes*- (Mañas and Pagán, 2005; Condón et al., 2011). However, the high resistance of some strains of *E. coli* -higher than that of *L. monocytogenes* cells- indicate that factors other than envelope structure probably also play a very relevant role in HHP resistance (Somolinos et al., 2008b; Espina et al., 2013; Ramos, 2016). Similarly, although it has often been reported that Gram-positive bacteria generally display greater UV and PEF resistance than Gram-negatives -and the high resistance of *L. monocytogenes* to these technologies seem to support this assumption- there are plenty of exceptions to this rule. Thus, microbial resistance to PEF would be determined by many factors, including size and shape, envelopes structure and some others that still have not been elucidated (Qin et al., 1991, 1998; Kehez et al., 1996; Álvarez et al., 2006a; Somolinos et al., 2010b). Increased bacterial resistance to UV would probably be due to a series of factors which -apart from cell wall thickness- might also include cell size, pigmentation, composition, size and conformation of the genetic material, and DNA repair efficiency (Gayán et al., 2014a). Furthermore, as discussed above, the variability in UV resistance among species and strains is larger than the divergences among genera, which makes it impossible to draw general conclusions (Gayán et al., 2014a).

Other interesting conclusions can be drawn from these results. On the one hand, as previously pointed out by Rodríguez-Calleja et al. (2006) and Cebrián et al. (2007), these results show that microorganisms which are the most resistant to a given stress are not necessarily more resistant to other types of stresses. Thus, for instance, whereas *S. aureus* was the most resistant microorganism to HHP and MS, it was among the less resistant to UV and PEF. Similarly, it is worth mentioning that, whereas the heat resistance of *S. senftenberg* 775 W is 10–100 times greater than that of all other species, its resistance to MS, PEF or UV and even HHP lies approximately in mid-range (Mañas et al., 2000b, 2001b, Álvarez et al., 2003a, 2006b; Gayán, 2014; Ramos, 2016). This finding can be easily explained by the varied modes of action of the four different technologies under review in this paper. On the other hand, and summarizing (parece necesario porque esto esta ya dicho arriba) they indicate that intra and inter-specific differences in resistance are very low for MS and, especially, for UV; they are medium-large for HHP and PEF, and very large for heat (**Table 2**). Furthermore, the fact that intra-specific differences in resistance to some agents (such as heat or UV) might be greater than inter-specific differences implies that, as recommended by the Environmental Protection Agency Scientific Advisory Panel for UV Water Disinfection (Oteiza et al., 2010), strains, and not species, should be used as an indicator to establish process criteria for these technologies. Alternatively, a cocktail of strains of each pathogen should be used.

**Table 2:** Factors affecting the resistance of bacterial foodborne pathogens to heat, MS, PEFs, HHP, and UV and degree of influence.

		Heat	MS	PEF	HHP	UV
Intrinsic factors	Inter-species variation	Very large	Low	Large-very large	Large	Very low
	Intra-species variation	Large-very large	Low	Low-medium	Medium-large	Very low
Process factors	Specific	Treatment time	Amplitude Pressure Specific energy Treatment time	Electric field Strength Pulse size/shape Specific energy Treatment time	Pressure Treatment time	Dose (J/ml)
	Temperature	Very large	Low-medium	Large	Large	Low-medium
Pre-treatment factors	Growth phase	Very large	Low	Medium-large	Large	Very low
	Growth temperature	Medium-large	Very low	Very low-low	Medium	Very low
	Prior stresses	Large	Very low	Low	Very low-low	Very low
Product parameters	pH	Large	Very low	Large	Large	Very low
	a_w_	Large	Low	Medium	Large	Very low
	Composition and others	Very large	Low	Medium-large	Large	Large
Recovery conditions	Sublethal injury?	Yes	No	Yes	Yes	No

*Very large: >100-fold variation. Large: 10–100-fold variation. Medium: 5–10-fold variation. Low: 2–5-fold variation. Very low: <2-fold variation.*

## Factors Affecting the Resistance of Bacterial Foodborne Pathogens to MS, PEF, HHP and UV

### Factors Acting Prior to Treatment

#### Influence of the Physiological State of the Microbial Cells

The influence of the type of microorganism, the species, and the strain under investigation on microbial resistance to novel food processing technologies has been well-documented. Nevertheless, growing evidence suggests that the cell’s physiological state might be just as important: it conditions the expression of resistance and repair mechanisms and thereby determines the degree of resistance that a cell will display. In other words, each strain possesses a gene pool coding for different resistance systems, but the cells’ physiological state decisively determines which of those resistance systems will be expressed, as well as their degree of expression.

**FIGURE 2:**
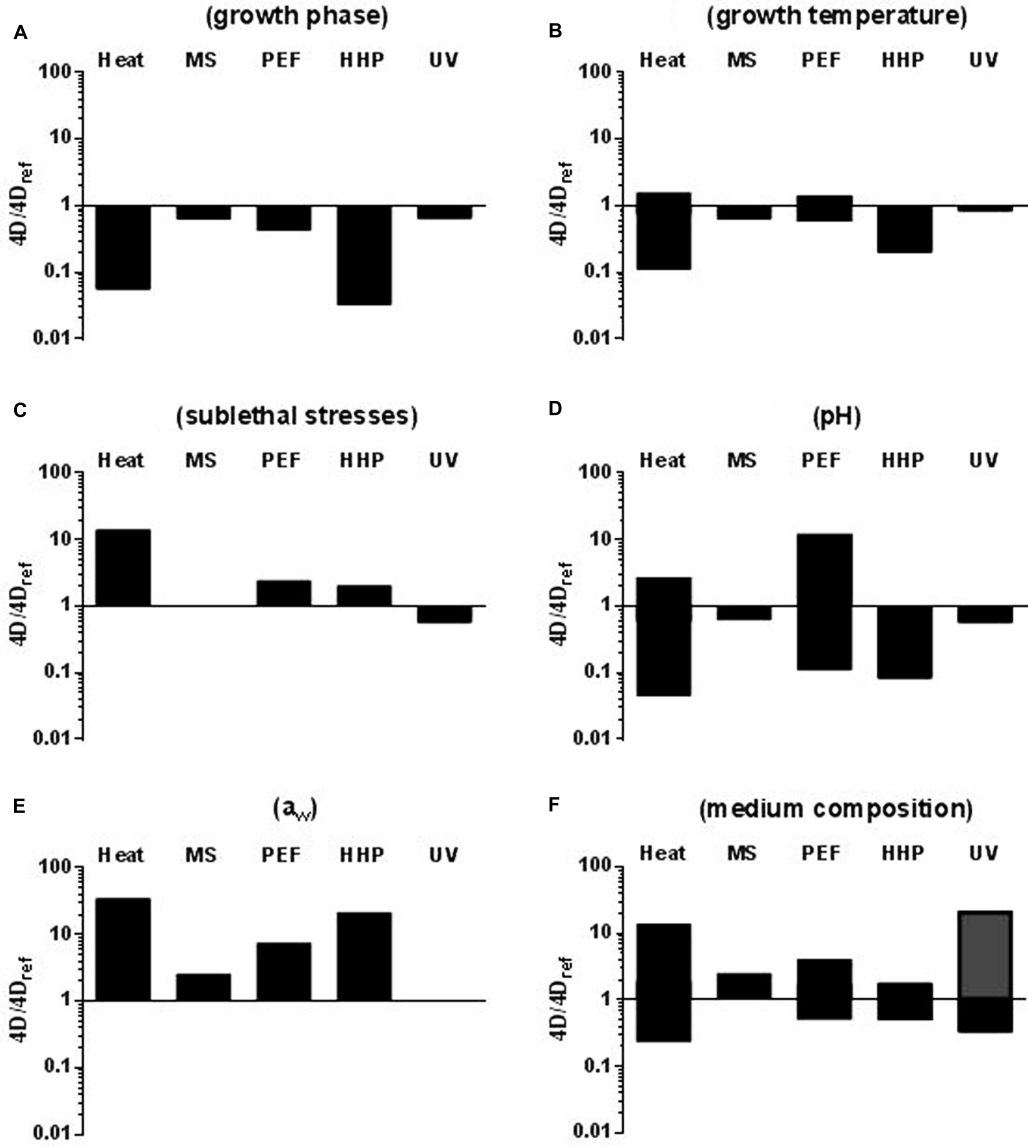
**Influence of different factors on microbial resistance to heat, MS, PEF, HHP, and UV. (A)** Growth phase (exponential vs. stationary); *4D_ref_*: stationary growth phase cells. **(B)** Growth temperature; *4D*_ref_:cells grown at 37°C. **(C)** Exposure to sublethal shocks; *4D_ref_*: non-stressed stationary growth phase cells. **(D)** Treatment medium pH (7.0 vs. 4.0); *4D_ref_*: cells treated at pH 7.0. **(E)** Treatment medium a_w_ (>0.99 vs. 0.96); *4D_ref_*: cells treated in media of a_w_ = >0.99. **(F)** Treatment medium composition; *4D_ref_*: cells treated in buffer. Bars indicate the maximum difference in *4D* values reported. In figure F the gray bar indicates the increase in microbial resistance to UV caused by an increase in the absorptivity of the medium (see text). See text for references.

Among all determinant factors, the physiological state of the bacterial cell growth phase is probably the one which has attracted the most attention. Exponential growth phase cells have proven to be less resistant to the four technologies studied herein than stationary growth phase ones (Cebrián et al., 2007, 2009, 2010a; Somolinos et al., 2008a; Arroyo et al., 2010a,b, 2011a, 2012b; Gayán et al., 2011, 2012c, 2014b, 2015). This has also been demonstrated for heat (Cebrián et al., 2007, 2009; Arroyo et al., 2009), and for both Gram-positive and Gram-negative species (**Figure 2A**). However, whereas entry into stationary growth phase did not lead to an increase in MS, UV, or PEF resistance (*4D* values) higher than 2.3-fold for any of the microorganisms investigated, it supposed an increase in HHP resistance of up to 31 times for *C. sakazakii* (Arroyo et al., 2011a). It should be noted that the magnitude of the change in baro-resistance brought about by a change in growth phase is greater than the intra- and even inter-specific differences in resistance. Thus, for instance, *E. coli* stationary growth phase cells would be more HHP resistant than *S. aureus* exponential growth phase ones, thereby implying that, in a product containing these two types of cells, *E. coli* cells would be regarded as the target microorganism. Although this certainly applies theoretically, one should point out that it is very unlikely that such a scenario would occur in real food products.

On the other hand, data accumulated seem to indicate that the influence of growth temperature and pre-exposure to environmental stresses on microbial survival to novel food preservation technologies would be much lower than that of growth phase and also lower than to heat (**Figures 2B,C**). Thus, for instance, whereas up to a 10-fold increase in thermo-resistance, approximately, has been found for *E. coli* after increasing growth temperature from 10 to 42°C (Cebrián et al., 2008) increasing growth temperature from 10 to 37°C resulted in a fivefold change in *4D_HHP_* values for *C. sakazakii* (Arroyo et al., 2011a). Varying growth temperature has even a less pronounced influence on microbial resistance to MS, PEF and UV. Thus, a maximum of threefold change in the *4D_PEF_* value was found by Cebrián et al. (2008) for *E. coli* cells but no significant differences were found in PEF resistance for *S. aureus* (Cebrián, 2009) or for *L. monocytogenes* (Álvarez et al., 2002), in MS resistance for *L. monocytogenes* or *S. typhimurium* (Pagán et al., 1999a; Condón et al., 2011) or in UV resistance for *C. sakazakii* (Arroyo et al., 2012b) or *E. coli* (Gayán, 2014), regardless of the growth temperature. From the limited information available concerning the development of resistance responses that could increase bacterial survival to non-thermal technologies such as MS, PEF, HHP, and UV (Cebrián et al., 2012) it can be concluded that the exposure of bacterial cells to sublethal stressing conditions capable of triggering homologous resistance responses (acid and alkaline pH, hydrogen peroxide, osmotic heat, and cold shocks) has an effect on microbial resistance to novel technologies much lower than that reported for heat treatments (Somolinos et al., 2008a; Cebrián et al., 2010b, 2012; Arroyo, 2011; Arroyo et al., 2012a; Gayán, 2014; **Figure 2C**).

A discussion of possible explanations for these results would lie beyond the scope of this article. However, it is worthwhile to mention that in many bacterial species the increased stress resistance of stationary growth phase cells as compared to exponential growth phase cells has been partly attributed to the induction of alternative general stress sigma factors (Abee and Wouters, 1999). General stress sigma factors include sigma S, also known as rpoS, in Gram-negative bacteria, and sigma B in Gram-positive bacteria, which are considered by many researchers as functionally homologous (Gertz et al., 2000; Hengge-Aronis, 2000). According to our data (Somolinos et al., 2008a, 2010a; Cebrián et al., 2009; Gayán et al., 2014b), the deletion of sigma factors resulted in a significant decrease in resistance to all the technologies here reviewed, but the differences between parental and isogenic Δ*sigB* or Δ*rpoS* mutants were much smaller for PEF, MS, and UV than for heat and HHP. These results strongly suggest that growth phase would have a higher impact on microbial survival to those technologies for which general stress response plays a more relevant role. Similarly, since the development of cross-resistance responses is also generally linked to the induction of these general stress responses, it is reasonable to think that development of cross-resistances to these technologies would be less generalized among bacteria and would probably have a lower impact. Nevertheless, it should be noted that certain relevant exceptions have been documented, such as the development of PEF resistance in *S. aureus* after heat and alkaline shocks (Cebrián et al., 2012), or the increased resistance of heat-shocked *E. coli* cells to HHP (Aertsen et al., 2004). On the other hand, only for HHP a relationship between the expression of heat shock proteins and microbial resistance has been observed (Aertsen et al., 2004) and the role of membrane fluidity on bacterial resistance to MS, PEF, HHP, and UV is still a matter of debate. These findings might explain the different influence that growth temperature has on microbial resistance to the four technologies here reviewed.

There is a very limited amount of information regarding the effect of other factors acting prior to the treatment, such as growth medium pH or atmosphere, on microbial resistance to these technologies and further research on this topic would be required and very useful. In any case, what these results clearly point out is that, when one is determining the target microorganism for a particular technology, one should certainly consider the possible influence of growth conditions and of exposure to stressing agents on microbial resistance.

### Factors Acting During Treatment

Factors acting during treatment can be classified in two groups: (1) medium properties and (2) processing factors.

#### Medium Properties

As highlighted above, microbial resistance to any inactivation agent depends on the cell’s physiological state. However, it is also influenced by a multitude of environmental factors that come into play in the course of treatment. Among the environmental factors affecting microbial resistance, the most investigated are pH, water activity, and chemical composition of the medium.

##### Treatment medium pH

pH is one of the environmental factors with the greatest influence on microbial resistance to heat (Tomlins and Ordal, 1976; Jay, 1992; Mañas et al., 2003; Arroyo et al., 2009), HHP (Mackey et al., 1995; Stewart et al., 1997; Alpas et al., 2000; Koseki and Yamamoto, 2006; Arroyo et al., 2009) and PEFs (Álvarez et al., 2000, 2002; Aronsson and Ronner, 2001; Geveke and Kozempel, 2003; García et al., 2005a,b, 2007; Saldaña et al., 2010a,b). Acidification is easily modifiable in foodstuffs and is frequently applied in the food industry. Treatment medium pH hardly affects microbial resistance to MS and UV, as opposed to other technologies. As **Figure 2D** shows, a reduction of pH from 7.0 to 4.0, which can lead to a 22-fold reduction of the *4D* values to heat and 12-fold to HHP (Arroyo et al., 2009, 2011a), only reduces the *4D_MS_* values by 1.6 times and does not result in a significant change of *4D*_UV_ values.

The influence of treatment medium pH on microbial resistance to PEF has attracted the interest of the scientific community for many years, since it differs widely from the way it affects microbial resistance to other technologies. Thus, in general terms, reducing treatment medium pH results in a decrease in the PEF resistance of Gram-positive cells (García et al., 2005a,b, 2007; Saldaña et al., 2010a). Conversely, decreasing treatment medium pH to a value of 5.0–5.5 also results in a decrease in PEF resistance for Gram-negative cells but further decreases (to pH 3.5–4.0) have the opposite effect (an increase in PEF resistance; García et al., 2005a,b, 2007; Saldaña et al., 2010b, 2012; Somolinos et al., 2010b). The latter increase is only observed if organic acids are added or present; thus, the type of acid is of essential importance (Somolinos et al., 2010b). This finding is of the highest relevance, since it implies that PEF pasteurization of low-pH food products -such as juices- should target Gram-negative pathogens.

It should also be noted that, according to our data (and also to that of some other authors, cf. Alpas et al., 2000), the baroresistance at acid pH of *E. coli* strains would be comparable to that of *S. aureus*, as previously pointed out for neutral pH. Given the higher acid tolerance of *E. coli* and the inability of *S. aureus* to synthesize enterotoxins at pH below 4.5 (ICMSF, 1996), *E. coli* should be considered the target microorganism for HHP in these types of products.

At present, the mechanism of Gram-positive bacterial sensitization to HHP and PEF (in this case only regarding Gram-positive bacteria) when treated at low pHs is not accurately known. It has been suggested that loss of membrane continuity would impair pH homeostasis, which could modify the intracellular pH affecting main components of the cell (DNA, RNA, enzymes, etc.; Vega-Mercado et al., 1996; Pagán et al., 2001). On the other hand, in spite of the difficulty of envisioning what kind of interaction between organic acid molecules and cell structures would have the capability of protecting Gram-negative cells against the action of PEF, the data obtained suggest that such an interaction would probably have something to do with the outer membrane. According to Somolinos et al. (2010b), the repair mechanisms of Gram-negative cells in the presence of organic acids at pH 4.0 are either are more efficient, or the membrane injuries caused by PEF are less severe and more easy to repair under favorable conditions. Finally, if treatment medium pH is a factor having little or no influence on microbial resistance to MS and UV, this can be explained by those two technologies’ specific mechanisms of inactivation.

##### Treatment medium water activity (a_w_)

Conversely to treatment medium’s pH, a reduction of its water activity usually results in an increase in microbial resistance to most food preservation technologies. Furthermore, according to published data, water activity is the parameter that exerts the greatest influence on microbial resistance to heat, PEF and HHP (**Figure 2E**). Thus, for instance, it has been demonstrated that reducing the water activity in the treatment medium can lead to a several 100-fold increase in bacterial resistance to heat (Kwast and Verrips, 1982; Sumner et al., 1991). According to our data, reducing water activity from >0.99 to 0.96 can increase the *4D*-values of heat by more than 30 times (Álvarez et al., 2003b, 2006b; Arroyo et al., 2009). The same change in water activity also led to increases greater than 10-fold in the PEF and HHP *4D*-values for *C. sakazakii* (Arroyo et al., 2010a, 2011a) but only to a less than threefold increase in the *4D*_MS_ values of *S. enterica* and *C. sakazakii* (Álvarez et al., 2003b, 2006b; Arroyo et al., 2010b); this change in water activity does not influence the *4D*_UV_ values of any of the species investigated by Gayán et al. (2011, 2012b, 2012b, 2015) and Arroyo et al. (2012b).

The molecular mechanisms involved in the acquisition of heat resistance by bacteria treated in low *a*_w_ media are not yet clear, although the following factors have been suggested: a dehydration of the cytoplasm followed by cell shrinkage, a reduction in pore size and a decreased loss of intracellular compounds (Gibson, 1973), or a stabilization of proteins and enzymes resulting from the formation of intramolecular links (Hansen and Riemann, 1963). It has also been proposed that the interaction of trehalose with membrane phospholipids could stabilize the membrane (Crowe et al., 1984). Similarly, overall cell volume reduction might also explain the increase in PEF resistance, since the electroporation threshold is dependent on cell size (Álvarez et al., 2006a). Cell shrinkage could likewise probably lead to a thickening of the cell membrane, followed by a reduction of membrane permeability and fluidity: both of these phenomena are supposed to increase microbial resistance to PEF (Neidhardt et al., 1990). Similar mechanisms have been proposed to explain the increase in microbial resistance to HHP. It has been demonstrated that the increased heat resistance observed in media of low water activity is partly due to a stabilization of cell structures against heat, and partly due to a higher capacity to repair the damages inflicted by heat (Álvarez et al., 2003b). By contrast, increased microbial resistance to PEF and HHP does not seem to be related to an increase in the ability to repair sublethal damages (Arroyo et al., 2010a, 2011a). Since microbial inactivation by MS at low water activities is an “all or nothing” event, this might partly explain the factor’s comparatively low influence on resistance to MS. Finally, as pointed out previously for pH, is seems logical that reducing the water activity of the media would not have an influence on microbial UV resistance, given the specific mode of action of this agent on DNA.

The protective effect of low a_w_ media on microbial inactivation by HHP and PEF has been proven to depend on the solute added. Thus, at the same level of water activity, microbial cells tend to be more pressure-sensitive in glycerol than in monosaccharides and disaccharides (Patterson, 2005). Similarly, salt is generally less protective against HHP than carbohydrates (Smelt, 1998). Regarding PEF, it has been reported that microorganisms are more sensitive to PEF when glycerol is added to the treatment medium than when the solute added is sucrose (Álvarez et al., 2006a).

##### Treatment medium composition

It is well-known that microbial resistance to most technologies changes with the composition of the treatment medium (Tomlins and Ordal, 1976; Hülsheger et al., 1981; Patterson et al., 1995; Grahl and Märkl, 1996; Simpson and Gilmour, 1997; Hauben et al., 1998; Mañas et al., 2001a,b; Gayán et al., 2014a). Although, it has been suggested that these changes in resistance could be due to pH and/or to water activity differences, many authors have demonstrated that microorganisms can display a differing degree of heat, PEF and HHP resistance in several types of media featuring the same pH and/or a_w_ (Baird-Parker et al., 1970; Corry, 1974; Hülsheger et al., 1981; Condón and Sala, 1992; Patterson et al., 1995; Grahl and Märkl, 1996; Simpson and Gilmour, 1997; Hauben et al., 1998; Mañas et al., 2001b). One could therefore conclude that certain chemical components, regardless of pH and water activity, might protect bacterial cells against different food preservation technologies. In some occasions, however, the opposite effect has also been observed (Arroyo et al., 2011a; Gayán, 2014; Serrano, 2016).

**Figure 2F** depicts the maximum influence of medium composition on microbial resistance to heat, MS, PEF, HHP, and UV. In order to elaborate this figure, we compared the *4D* values obtained for different microorganisms suspended in different food products and exposed to the four technologies in buffer of similar (if not equal) pH and a_w_. Thus, the changes in microbial resistance here reported would be due to the food product’s specific composition and cannot be attributed to its pH or a_w_.

According to our data, the influence of medium composition on microbial resistance to MS, PEF and HHP is much lower than to heat. Thus, MS resistance barely changes in laboratory media and liquid foods such as milk, juices, vegetable soups and liquid whole egg (Mañas et al., 2000b; Arroyo et al., 2010b, 2011b,c; Condón et al., 2011). Similarly, microbial HHP resistance hardly increases threefold (Arroyo et al., 2010a); among all products studied (including milk, juices, and liquid whole egg), and only milk induced in a remarkable increase in the *4D* PEF values -estimated as more than fourfold (García et al., 2005c; Monfort et al., 2010; Arroyo, 2011)-. It should be noted that other authors have reported greater differences in HHP resistance due to changes in medium composition (Patterson, 2005). By contrast up to a 14-fold increase in heat resistance has been reported for *C. sakazakii* when treated in apple juice as compared to a buffer of the same pH and a_w_ (Arroyo et al., 2009). Protective effects against heat of different food products such as liquid egg, milk, juices and vegetable soups have been also documented (Mañas et al., 2000b, 2001b; Arroyo et al., 2009; Serrano, 2016). In should be noted that, the opposite effect -a decrease in heat resistance when treated in food- has also been sporadically observed (Serrano, 2016).

The case of UV is quite particular. Factors other than the optical properties of the medium have a very low influence on microbial resistance (Gayán et al., 2014a). Conversely, as pointed out by Koutchma et al. (2009), the most influential product characteristics related to the lethal efficacy of UV technologies are optical properties, mainly the UV absorbance and the turbidity of the medium. Thus, color components, soluble compounds, and suspended solids can absorb, reflect, and scatter incidental light, thereby reducing the number of photons available for killing microorganisms (Koutchma et al., 2009). According to Gayán et al. (2011) an increase in medium’s absorptivity of 15.92 cm^-1^ leads to a 10-fold increase in the *4D* values for *E. coli*. Similar results were obtained for other microorganisms (Gayán et al., 2012c, 2014b, 2015).

At present, the mechanisms involved in these increases and/or decreases in microbial resistance are not accurately known. Milk is one of the few products for which the underlying mechanism leading to the change in resistance has been studied in depth. Thus, it has been proposed that the increased microbial resistance to PEF and HHP when treated in milk would be probably due to the stabilization effect of divalent cations on cell membranes (Hauben et al., 1998; Álvarez et al., 2006a).

#### Processing Factors

Since most processing factors (listed in **Table 2**) are specific to each technology, they cannot be compared across the board. Therefore, the only factor in this group which we will discuss is treatment temperature.

**FIGURE 3:**
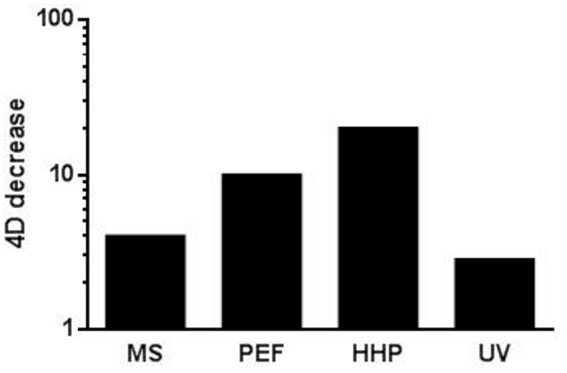
**Influence of an up-shift in treatment temperature (from ambient temperature to 55°C -for HHP from ambient to 50°C-) on the *4D* values calculated for the different technologies reviewed.** See text for references.

Although all the novel technologies reviewed herein are regarded as non-thermal, various authors have proposed to combine them with sublethal or even lethal temperatures in order to increase the process’s overall lethality (Sala et al., 1992; Patterson and Kilpatrick, 1998; Raso et al., 1998d,e; Heinz et al., 2003; Raso and Barbosa-Cánovas, 2003; Leadley, 2005; Patterson, 2005; Álvarez et al., 2006a; López-Pedemonte et al., 2006; Saldaña et al., 2010c, 2012; Gayán et al., 2011, 2012a,b,c, 2014b, 2015). A combination with sublethal temperatures has proven to enhance the lethal effect of MS (Sala et al., 1992; Raso et al., 1998d,e; Pagán et al., 1999a,b,c; Arroyo et al., 2011b,c), PEF (Álvarez et al., 2006a; Cebrián, 2009; Saldaña et al., 2010c, 2012) and of UV-C (Gayán et al., 2011, 2012a,b,c, 2014b, 2015; Arroyo et al., 2012b). On the other hand, treatment temperature can also have a significant effect on microbial resistance to HHP. Thus, over a particular temperature threshold, increasing treatment temperature also leads to an increase in HHP lethality (Patterson, 2005; Ramos, 2016). Thus, the combined application of these novel technologies with moderate temperatures appears to be one of the most interesting alternatives for developing combined processes since, as can be observed in the figure, an increase in treatment temperature from ambient temperature to 55°C leads to significant decreases in *4D*-values in the case of all four technologies, including >10-fold decrease for PEF and HHP (**Figure 3**). It should be noted that most of the data reported seem to indicate that the magnitude of the increase in lethality by increasing treatment temperature seems to be greater for PEF and HHP than for MS and UV. These results are in concordance with a further finding: whereas increases in temperature within the physiological range (e.g., from 25 to 40°C) have proven to increase the lethality of PEF and HHP, more elevated temperatures (close to 50°C or even higher) are required to induce a significant decrease in microbial resistance to MS and to UV (Raso et al., 1998d,e; Pagán et al., 1999a,b,c; Tassou et al., 2008; Cebrián, 2009; Saldaña et al., 2010c; Arroyo et al., 2011b,c; Gayán et al., 2011, 2012a,b,c, 2014b, 2015).

According to most authors, the increased sensitivity of bacterial cells to MS, PEF, and HHP when treated at sublethal temperatures would probably be due to certain temperature-induced changes within the cell envelopes which might make them more vulnerable to mechanical stress (Sonoike et al., 1992; Pagán et al., 1999b; Álvarez et al., 2006a). Thus, it has been hypothesized that membrane fluidization of bacterial membranes would make them more sensitive to these three technologies (Stanley, 1991; Casadei et al., 2002; Condón et al., 2005). However, a number of researchers have pointed out that fluidization alone cannot adequately explain all the results reported, and that further factors must play a role in the temperature-dependent sensitization of bacterial cells to MS, HHP, and PEF (Casadei et al., 2002; Condón et al., 2005; Cebrián, 2009). Regarding UV, Gayán et al. (2013b) suggested that the synergistic lethal effect of UV-H treatments would be due to the inhibition of DNA excision repair resulting from membrane fluidification caused by simultaneous heating (Gayán et al., 2013b).

### Factors Acting After Treatment

Recovery conditions are generally acknowledged as one of the pivotal factors in microbial survival following exposure to a lethal agent (Mañas and Pagán, 2005). As Mackey (2000) has described, micro-organisms surviving the lethal action of preservation agents may be sublethally injured. This means that they might be able to repair the damage and outgrow, but only if environmental conditions are suitable. Thus, the final number of viable microbial cells after a particular treatment would be highly conditioned by recovery conditions, at least for those technologies that produce sublethal injuries in cells.

As pointed out by Mañas and Pagán (2005), the occurrence of sublethal injury has two main consequences. First, since injured cells might not be detected when selective conditions are used to enumerate survivors, an inadequate choice of recovery conditions can lead to an overestimation of the treatment’s lethality. Secondly, if repair is adequately prevented, the cell might not be able to outgrow damage, and the inactivation level attained might thus be more elevated. This opens up the possibility of developing new combined processes based on the use of these technologies in conjunction with additional preservation agents (hurdles) capable of interfering with cellular homeostasis maintenance.

All the technologies here reviewed -with the exception of MS- can lead to the appearance of sublethally injured cells (Pagán et al., 1999b; García et al., 2005a,b, 2007; Somolinos et al., 2008a,b, 2010a; Cebrián et al., 2009, 2010a; Arroyo et al., 2010a,b, 2011a,b; Saldaña et al., 2010a,b,c; Gayán et al., 2013b). However, given that these technologies’ mechanisms of action differ quite radically, the types of inflicted damage vary widely. Furthermore, the factors reviewed above -including the type of microorganism, its physiological state and the treatment conditions- also determine the types and severity of injuries caused. Consequently, the proportion of sublethally injured cells following treatment also varies widely depending on the agent, the microorganism and treatment conditions.

Sublethal damages to the cytoplasmic membrane have been observed following microbial exposure to PEF and HHP (García et al., 2005a,b, 2007; Somolinos et al., 2008a,b, 2010a; Cebrián et al., 2009, 2010a; Arroyo et al., 2010a, 2011a; Saldaña et al., 2010a,b,c). Likewise, sublethal damages to the outer membrane have been documented after having exposed microbial cells to HHP and, occasionally, to PEF (Somolinos et al., 2008b; Arroyo et al., 2010a, 2011a). Conversely, sublethal oxidative damages have been detected after HHP treatments (Aertsen et al., 2004; Cebrián et al., 2010a) but not after PEF. Further work would be required in order to fully characterize how the type of microorganism, its physiological state and the treatment conditions determine the proportion of sublethally injured cells after a treatment; still, at this point we can already rule out certain general trends. For instance, the proportion of sublethally damaged cells following PEF as well as HHP treatments seems to be higher for stationary growth phase cells than for exponential ones (Somolinos et al., 2008a,b; Cebrián et al., 2009, 2010a). Regarding PEF, the proportion of sublethally damaged cells has proven to be larger when treated at pH 7.0 than at pH 4.0 for Gram-positive cells and the opposite applies to Gram-negative cells (García et al., 2005a,b, 2007; Arroyo et al., 2010a; Saldaña et al., 2010a,b). Finally, increased microbial resistance to PEF brought about by decreasing the a_w_ of the medium does not seem to have any connection with a variance in the proportion of sublethally damaged cells (Arroyo et al., 2010a).

On the other hand, contradictory results have been obtained regarding the proportion of sublethally injured cells following HHP treatments at different pH levels: Condón et al. (2011) reported that the proportion of sublethally injured *S. aureus* cells was lower at acid pH than at neutral pH, but no difference in such proportions was found by Arroyo et al. (2011a). Although data are very scarce, increased microbial resistance to HHP caused by decreasing the a_w_ of the medium, as in PEF, does not seem to be related to a change in the proportion of sublethally damaged cells (Arroyo et al., 2011a). Among all these scenarios, the most relevant ones are those in which increased microbial resistance is associated with the appearance of an increased proportion of sublethally injured cells (e.g., the inactivation of Gram-negatives by PEF in acidic media), since they open up the possibility of developing combined processes capable of inactivating microorganisms under circumstances that could not be produced by the technology alone.

Many combinations of HHP and PEF with different agents such as lysozime, nisin, pediocin AcH, lacticin, lactoferrin, lactoferricin, EDTA, triethil citrate, essential oils, citral, carvacrol or limonene, all leading to increased microbial inactivation, have been described (Kalchayanand et al., 1994; Hauben et al., 1998; Somolinos et al., 2008a; Arroyo et al., 2010c; Monfort et al., 2012; Saldaña et al., 2012; Espina et al., 2013, 2014). It is also worth mentioning that another procedure has been proposed to achieve the inactivation of sublethally injured cells caused by HHP and PEF: namely, cells’ subsequent storage in acid media at refrigeration temperatures (García et al., 2005c; Somolinos et al., 2008a). This procedure is of special interest in the field of pasteurized juice processing, since it would not require the addition of any additional step or agent following HHP or PEF treatment.

Developing a combined procedure with UV based on the same principle seems more complex at first, since such a procedure should be based on the prevention of either light-dependent or light-independent DNA repair mechanisms (Gayán et al., 2013a). In this regard, preventing the exposure of treated cells to visible light might represent an alternative. However, the efficacy thereof seems to vary widely depending on the type of microorganisms (Gayán, 2014). On the other hand, as explained above, Gayán et al. (2013b) demonstrated that the increased lethality of UV treatments when applied at sublethal temperatures is due to the reduced ability of microbial cells to repair DNA caused by the fluidification of the membranes. Thus, this combined process once more illustrates how the prevention of microbial damage repair could increase the efficacy of treatments involving new preservation technologies.

## Concluding Remarks

Among the six bacterial foodborne pathogens here considered, *S. aureus* is the most resistant foodborne pathogen to MS and HHP and *L. monocytogenes* to UV. The target microorganism of PEF would change depending on the treatment medium pH. Thus, *L. monocytogenes* is the most PEF resistant microorganism at neutral pH but Gram-negatives (*E. coli*, *Salmonella* spp., *C. sakazakii, C. jejuni*) would display a similar or even higher resistance at acidic pH. It should be noted that, in acidic products, the baroresistance of some *E. coli* strains would be comparable to that of *S. aureus*.

Microbial resistance to MS, PEF, HHP, and UV depends on many factors including the type of microorganism, its physiological state, and treatment and recovery conditions. However, the influence of these factors on microbial resistance to each technology varies widely. In general the factors reviewed have a greater impact on bacterial resistance to HHP and PEF than to MS and UV. Thus, inter- and intra-specific differences in microbial resistance to PEF and HHP are much greater than differences in resistance to MS and, especially, to UV. It should be remarked that, in some cases, intra-specific differences in resistance exceed inter-specific ones (e.g., for UV). This is highly relevant when one is determining the target microorganism, for instance.

Among all factors acting prior to treatment, the one with the greatest impact on microbial resistance is the growth phase, particularly in relation with treatments with HHP. However, the role played by other factors, such as the development of cross-resistance responses, should not be overlooked. Both the pH and a_w_ of the treatment medium highly condition microbial resistance to PEF and HHP but not to MS and UV. The magnitude of this change in microbial resistance is even greater than the inter-specific differences in resistance. This fact leads to a change on the target microorganism of PEF depending on the pH of the treatment medium. On the other hand, the optical properties of the medium are, by far, the most influential product characteristics in terms of the lethal efficacy of UV technologies.

An increase in treatment temperature is regarded as one of the most promising methods to increase the lethality of MS, PEF, HHP and UV treatments, and to facilitate their industrial implementation. As described above, an increase in treatment temperature leads to a significant increase in lethality of the four technologies here reviewed. The appearance of sublethally damaged cells following PEF and HHP treatments could also be exploited in order to design combined processes. Further work needs to be carried out if we want to fully characterize microbial resistance to these combined processes, since the procedures themselves might cause changes in the target species or strains.

Regarding the use of these technologies as alternatives to heat for pasteurization purposes the practical implications of the conclusions presented above is evident. Both MS and UV light share in common that its lethality hardly changes with the species and the environmental factors -but for optical properties and UV-. Thus, both would be especially interesting as an alternative to heat for preservation of thermal-sensitive liquid foods, especially when raw material is contaminated with very heat-resistant bacterial species, or when food components protect microorganisms to heat. Furthermore, its combination with mild temperatures appears as a very attractive alternative for improving its lethal effect. On the other hand, both HHP and PEF inactivation is affected by many factors -and in some cases very drastically-. This might turn out to be an advantage in some cases, such as the pasteurization of acidic products by HHP. In any case, the development of combined processes (including heat or other preservation methods) appears as the most feasible approach for the design of pasteurization processes based on HHP or PEF.

If we want to exploit these technologies’ full potential, further research needs to be carried out in order to gain a more specific understanding of their mechanisms of action -especially those of PEF and HHP- and to exhaustively characterize the influence of all the factors acting before and during treatment. This would also prove thoroughly useful in the area of process optimization, and in the design of combined procedures with a sound scientific basis.

## Author Contributions

All authors listed, have made substantial, direct and intellectual contribution to the work, and approved it for publication.

## Conflict of Interest Statement

The authors declare that the research was conducted in the absence of any commercial or financial relationships that could be construed as a potential conflict of interest.
